# The Effect of Thiamine and Thiamine Pyrophosphate on Oxidative Liver Damage Induced in Rats with Cisplatin

**DOI:** 10.1155/2013/783809

**Published:** 2013-06-06

**Authors:** Mehmet Ibrahim Turan, Isil Siltelioglu Turan, Renad Mammadov, Konca Altınkaynak, Abdullah Kisaoglu

**Affiliations:** ^1^Department of Pediatrics, Faculty of Medicine, Ataturk University, Erzurum 25240, Turkey; ^2^Department of Internal Medicine, Pasinler State Hospital, Erzurum 25300, Turkey; ^3^Department of Pharmacology, Ataturk University, Erzurum 25240, Turkey; ^4^Department of Biochemistry, Erzurum Research and Educational Hospital, Erzurum 25070, Turkey; ^5^Department of Surgery, Ataturk University, Erzurum 25240, Turkey

## Abstract

The aim of this study was to investigate the effect of thiamine and thiamine pyrophosphate (TPP) on oxidative stress induced with cisplatin in liver tissue. Rats were divided into four groups; thiamine group (TG), TPP + cisplatin group (TPG), healthy animal group (HG), and cisplatin only group (CG). Oxidant and antioxidant parameters in liver tissue and AST, ALT, and LDH levels in rat sera were measured in all groups. Malondialdehyde levels in the CG, TG, TPG, and HG groups were 11 ± 1.4, 9 ± 0.5, 3 ± 0.5, and 2.2 ± 0.48 **μ**mol/g protein, respectively. Total glutathione levels were 2 ± 0.7, 2.8 ± 0.4, 7 ± 0.8, and 9 ± 0.6 nmol/g protein, respectively. Levels of 8-OH/Gua, a product of DNA damage, were 2.7 ± 0.4 pmol/L, 2.5 ± 0.5, 1.1 ± 0.3, and 0.9 ± 0.3 pmol/L, respectively. A statistically significant difference was determined in oxidant/antioxidant parameters and AST, ALT, and LDH levels between the TPG and CG groups (*P* < 0.05). No significant difference was determined between the TG and CG groups (*P* > 0.05). In conclusion, cisplatin causes oxidative damage in liver tissue. TPP seems to have a preventive effect on oxidative stress in the liver caused by cisplatin.

## 1. Introduction

Chemotherapeutic agents play the most important role in present-day cancer treatment. However, in addition to the benefits of their use, they can also cause side effects and toxicity. The severe side effects and toxicity of chemotherapeutics are the main limiting factors in cancer treatment [1].

Cisplatin is a platinum-based drug used as an antineoplastic agent. It has a wide spectrum of use in various tumoral events, including the lung, kidney, ovary, testis, bladder, head, neck, and endometrium [2]. Significant side effects of cisplatin can be seen in several important tissues. These side effects represent a significant obstacle to the effective treatment of cancer [3, 4]. One of the target organs for cisplatin-related toxicity is the liver [5]. Studies have reported that the production of reactive oxygen species and decreasing antioxidants in serum are involved in the development of cisplatin toxicity [6, 7]. It has been suggested that free radical-associated organ damage is the result of impairment of antioxidant defense mechanisms [8]. Cisplatin-related toxicity in tissues has been shown to be closely associated with increased lipid peroxidation [9]. Various mechanisms have been proposed regarding hepatotoxicity caused by cisplatin, but the cause is still not entirely clear [10]. Studies have particularly been performed on impairments in the oxidant/antioxidant balance associated with mitochondrial injury in cisplatin-associated liver toxicity, and various pharmacological agents to reduce oxidative stress have been investigated [10, 11]. Understanding the mechanism leading to toxicity will contribute to the development of new ways of preventing cisplatin-associated toxic effects. Research on the subject is therefore still continuing. The thiamine whose effects on cisplatin-induced hepatotoxicity are investigated in this study is a water-soluble vitamin, while thiamine pyrophosphate (TPP) is an active metabolite of thiamine. TPP forms as the result of thiamine in the liver reacting with the enzyme thiamine pyrophosphokinase. TPP catalyzes several chemical reactions in the body. Thiamine is used on the pentose phosphate pathway and increases antioxidant formation and NADPH levels [12]. Thiamine has been shown to increase lipid peroxidation in liver cells, and its antioxidant effect has been investigated [13]. However, TPP's antioxidant activity has been shown to be superior to that of thiamine and to be more protective against oxidative damage [14].

Our scan of the literature revealed no data regarding the prevention by thiamine and TPP of oxidative damage in the liver induced with cisplatin. The aim of this study was therefore to investigate whether thiamine and TPP were effective in preventing oxidative damage in the rat liver induced with cisplatin. 

## 2. Materials and Methods

### 2.1. Animals

Twenty-four male albino Wistar rats weighing 210–230 g were obtained from the Ataturk University Medicinal and Experimental Application and Research Center, Erzurum, Turkey. Animals were allowed 14 days to acclimatize before the experiments began. They were maintained in a 12:12 h light/dark cycle (lights on 07:00–19:00 h) in air-conditioned constant temperature (22 ± 1°C) colony room, with free access to water and 20% (w/w) protein commercial chow. This commercial chow contained 14% protein, 2% calcium, 1% sodium, 1% phosphorus, 10,000 IU/kg vitamin A, 1000 IU/kg vitamin D_3_, and 30 IU/mg vitamin E. Animal experiments were performed in accordance with the national guidelines for the use and care of laboratory animals and were approved by the local animal ethics committee of Ataturk University, Erzurum, Turkey.

### 2.2. Chemical Substances

Of the chemical substances used for the experiments, cisplatin CDDP vials (50 mg/100 mL; Cisplatin-Ebewe) were provided by Liba, Turkey. Thiamine (50 mg/mL) and TPP (50 mg/mL) were provided by Biopharma, Russia, and thiopental sodium was obtained from IE Ulagay, Turkey.

### 2.3. Pharmacological Procedures

The animals were randomly divided into four groups before the experimental procedures were initiated (thiamine 20 mg/kg group (TG), TPP 20 mg/kg group (TPG), healthy animal (HG), and control groups (CG)). Each group contained six animals. All doses were administered intraperitoneally (ip) as milligrams per kilogram. The TG was administered 20 mg/kg thiamine, and the TPG was administered 20 mg/kg TPP by the ip route [15, 16]. The CG was administered distilled water as solvent. One hour after drug administration the TG, TPG, and CG groups were given cisplatin in a 5 mg/kg dose by the ip route once a day for 14 days. The HG was given distilled water once a day during that period. At the end of the study period, all animals were sacrificed with a high dose of anesthesia (50 mg/kg sodium thiopental). In this study, we elected to use doses employed for thiamine and TPP based on the results of previous experimental studies of ours [17, 18]. The doses used in this study are not equivalent to those used by humans because rats have different metabolic rates [19]. Livers were extracted, and biochemical examination was performed. The results from the TG and TPG groups were compared with those from the CG and HG groups.

### 2.4. Biochemical Analysis

#### 2.4.1. AST, ALT, and LDH Measurements

Venous blood samples were collected into tubes without anticoagulant. Serum was separated by centrifugation after clotting and stored at −80°C until assay. Serum AST and ALT activities as liver function tests, and LDH activity as a marker of tissue injury, were measured spectrophotometrically on a Cobas 8000 (Roche) autoanalyzer using commercially available kits (Roche Diagnostics, GmBH, Mannheim, Germany).

#### 2.4.2. Biochemical Analysis of Liver Tissue

In this part of the study, 0.2 mg of whole liver tissue was weighed for each liver. The samples were homogenized in ice with 2 mL buffers (consisting of 0.5% HDTMAB (0.5% hexadecyl trimethyl ammonium bromide) pH: 6 potassium phosphate buffer for myeloperoxidase analysis, consisting of 1.15% potassium chloride solution for thiobarbituric acid reactions (TBARS) analysis, and pH: 7.5 phosphate buffer for the total glutathione analysis). They were then centrifuged at 4°C, 10,000 rpm, for 15 min. The supernatant part was used as the analysis sample. For all measurements, the tissue-protein estimation was performed according to the method described by Bradford [20].

*Malondialdehyde (MDA) analysis*: concentrations of liver tissue lipid peroxidation were determined using the TBARS, a modified version of the method described by Nabavi et al. [21].

*Myeloperoxidase (MPO) analysis*: MPO in the liver tissue was measured according to the method described by Wei and Frenkel, with some modifications [22].

*Total glutathione (tGSH) analysis*: tGSH in the liver tissue was measured according to the method described by Sedlak and Lindsay, with some modifications [23].

*Glutathione S-transferase (GST) activity* in liver tissue was determined using the method described by Habig and Jakoby [24].

*Glutathione peroxidase (GPx) activity* was determined according to the method described by Lawrence and Burk in liver tissue [25].

*Glutathione reductase (GR) activity* in liver tissue was determined according to Carlberg and Mannervik's method [26].

*Isolation of DNA from Liver Tissue*. Liver tissue was drawn and DNA isolated using Shigenaga et al.'s modified method [27].

#### 2.4.3. DNA Hydrolysis with Formic Acid

Approximately 50 mg of DNA was hydrolyzed with 0.5 mL of formic acid (60%, vol/vol) for 45 min at 150°C [28]. The tubes were allowed to cool. The contents were then transferred to Pierce microvials (Sigma Co., Munich, Germany), covered with Kleenex tissues (Kimberly-Clark, USA) cut to size (secured in place using a rubber band), and cooled in liquid nitrogen. Formic acid was then removed by freeze-drying. Before analysis using high-performance liquid chromatography (HPLC), they were redissolved in the eluent (final volume 200 *μ*L) [29, 30].

#### 2.4.4. Measurement of 8-Hydroxy-2-Deoxyguanosine (8-OH Gua) with High-Performance Liquid Chromatography (HPLC) System

The amount of 8-OH gua and guanine (Gua) was measured using an HPLC system equipped with an electrochemical detector (HP Agilent 1100 module series, E.C.D. HP 1049 A), as described previously [28, 31]. The 8-OH gua levels were expressed as the number of 8-OH gua molecules/10^5^ gua molecules [32].

### 2.5. Statistical Analysis

All data were subjected to one-way analysis of variance using Statistical Package for Social Sciences 18.0 (Armonk, NY, USA) software. Differences among groups were determined using the least significant difference option, and significance was set at *P* ≤ 0.05. The results are expressed as mean ± standard deviation. 

## 3. Results

Table 1 shows the levels of MDA, MPO, tGSH, GST, GPx, GR, and DNA damage product measured in liver tissue. When the TG, TPG, and HG groups' oxidative damage products were compared with the CG group, a statistically significant difference was determined between the CG group and the other three groups, particularly TPG and HG. The most pronounced decrease in the activities of enzymes protecting against oxidative damage was in the CG and TG groups. The only statistically significant difference between these two groups was in tGSH levels (*P* < 0.05). In terms of tGSH, GST, GPx, and GR levels, a statistically significant difference was determined between the TPG and HG groups and the CG group (*P* < 0.0001).

In terms of levels of 8-OHGua/Gua, a product of DNA damage, between the groups, no statistically significant difference was observed between the CG and TG groups (*P* > 0.05), while the difference between the TPG and HG groups was statistically significant (*P* < 0.0001).

**Figure 1:**
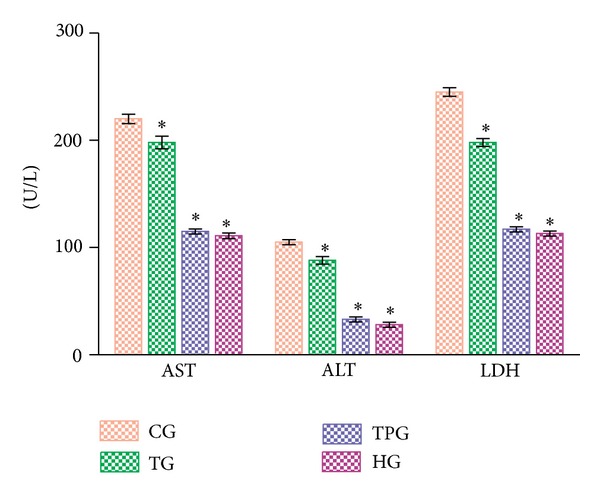
Comparison of groups in terms of serum AST, ALT, and LDH levels.*P < 0.0001, group data were compared against the CG group. Notes: differences among groups were obtained using ANOVA post hoc with the least significant difference option. Each group contained six animals. AST, ALT, and LDH levels are defined in U/L. Bars are mean ± standard deviation. CG, control group; TG, thiamine 20 mg/kg + cisplatin group; TPG, TPP 20 mg/kg + cisplatin group; CG, control group.

As shown in Figure 1, serum AST, ALT, and LDH levels in the CG group were 220 ± 4.3 U/L, 105 ± 2.4 U/L, and 245 ± 4 U/L, respectively, compared to 198 ± 5.9 U/L (*P* < 0.0001), 88 ± 3.5 U/L (*P* < 0.0001), and 198 ± 3.7 U/L (*P* < 0.0001) in the TG group; 115 ± 2.3 U/L (*P* < 0.0001), 33 ± 2.3 U/L (*P* < 0.0001), and 117 ± 2.4 U/L (*P* < 0.0001) in the TPG group. In the HG group, AST, ALT, and LDH levels were 111 ± 2.6 U/L (*P* < 0.0001), 28 ± 2.3 U/L (*P* < 0.0001), and 113 ± 2.3 U/L (*P* < 0.0001), respectively.

## 4. Discussion

This study investigated the effects of thiamine and TPP on oxidative liver damage induced with cisplatin in rats. The results show that TPP at 20 mg/kg significantly prevented oxidative liver damage induced with cisplatin, whereas thiamine at 20 mg/kg did not. As our results show, MDA concentrations in CG group rat livers were significantly higher compared to the HG group. MDA is the final product of lipid peroxidation. For various reasons, free oxygen radicals produced in excessive quantities affect membrane lipids containing unsaturated fatty acids more than other biomolecules. Interaction with membrane lipids leads to a rise in membrane permeability and severe cell damage [33]. Like MDA, MPO is an oxidant parameter and was significantly elevated in the CG group. Production of MPO by neutrophils and macrophages rises in areas of damage caused by various agents. MPO catalyzes reaction between hydrogen peroxide and chloride and produces the toxic compound hypochlorous acid. Hypochlorous acid is involved in the formation of hydroxyl radical (^−^OH) [34]. Studies have shown that MPO activity rises significantly compared to healthy tissue in liver tissue when oxidative damage is induced with cisplatin [35]. Elevated MDA and MPO levels in the CG group show that oxidative stress developed. Levels of MDA and MPO, products of oxidative damage, being particularly low in the TPG group, and the results being close to those of the HG group show that this stems from the antioxidant property of TPP. The information in the literature is therefore compatible with the results of our experiment [17, 18, 36, 37].

tGSH, GST, GPx, and GR levels were significantly lower in the CG group liver tissue compared to the HG group. Antioxidants suppress radical formation, repair oxidative damage, remove damaged molecules, and prevent mutations and reactive by-products [38]. tGSH, GST, GPx, and GR levels in liver tissue in the TPG group were significantly higher compared to the CG group, supporting our idea that TPP at a dose of 20 mg/kg plays a protective role against oxidative damage through various as yet unknown mechanisms. 

DNA damage has been determined to increase in tissue with elevated MDA and low tGSH levels [39]. TPP significantly prevented DNA oxidation in tissue with liver toxicity induced with cisplatin. The product reflecting DNA oxidation in tissue is 8-OHGua [40]. 8-OHGua is regarded as another DNA lesion and has been widely researched because of its known mutagenic effects [41]. Studies have shown that 8-OHGua levels in damage tissue rise in parallel to the rise in oxidant parameters and decrease in parallel with an increase in antioxidant parameters [42, 43]. This information from the literature agrees with our results. 

This investigation of the damage caused in the liver by cisplatin also evaluated enzymes associated with hepatocellular damage, such as serum AST, ALT, and LDH [44, 45]. Significantly elevated AST, ALT, and LDH levels were determined in the CG group, particularly in comparison to the TPG and HG groups. Previous studies have reported that cisplatin increases serum transaminases [11]. AST and particularly ALT are sensitive enzymes in showing hepatocyte damage [46]. Antioxidants have been reported to prevent excessive rises in AST, ALT, and LDH in hepatic injury [47, 48]. In this study, ALT in particular exhibited a 3-fold greater rise in the CG group compared to the HG group, while the rise in AST was 2-fold. This shows that hepatic cells are more affected in cisplatin-associated injury. A rise in hepatic enzymes was prevented in the TPG group. This result shows that TPP at a dose of 20 mg/kg plays a protective role against hepatic damage caused by cisplatin. 

At a general evaluation of the results, it is unclear why thiamine is not as effective in preventing cisplatin-related hepatotoxicity as TPP, its most important active metabolite. However, bearing in mind that TPP is formed from thiamine in hepatic cells, we think that cisplatin also has a negative impact on various enzymatic mechanisms involved in liver cells. This is because although thiamine is reported to be metabolized very quickly to TPP, there are as yet no established data on this, and the mechanism involved at the cellular level is unclear [49, 50]. In conclusion, cisplatin causes oxidative stress in the rat liver. This suggests that the use of TPP may be beneficial in preventing oxidative damage caused by cisplatin in the liver. 

## Figures and Tables

**Table 1:** Comparison of groups in terms of oxidant and antioxidants parameters in liver tissue.

	Groups			
	CG *n*: 6	TG *n*: 6	TPG *n*: 6	HG *n*: 6
MDA (*µ*mol/g protein)	11 ± 1.4	9 ± 0.5 *P* < 0.001*	3 ± 0.5 *P* < 0.0001*	2.2 ± 0.48 *P* < 0.0001*

MPO (U/g protein)	14 ± 2.1	12.5 ± 2.5 *P* > 0.05	4 ± 0.8 *P* < 0.0001*	3.1 ± 0.6 *P* < 0.0001*

tGSH (nmol/g protein)	2 ± 0.7	2.8 ± 0.4 *P* < 0.05*	7 ± 0.8 *P* < 0.0001*	9 ± 0.6 *P* < 0.0001*

GST (U/g protein)	5 ± 1.5	6.4 ± 1.4 *P* > 0.05	14 ± 1.5 *P* < 0.0001*	19 ± 1.9 *P* < 0.0001*

GPx (U/g protein)	7 ± 0.9	8.2 ± 0.7 *P* > 0.05	17 ± 0.6 *P* < 0.0001*	18.7 ± 1.9 *P* < 0.0001*

GR (U/g protein)	3 ± 0.6	3.7 ± 0.5 *P* > 0.05	11 ± 1.5 *P* < 0.0001*	14.5 ± 1.1 *P* < 0.0001*

DNA damage product 8-OHGua/Gua (pmol/L)	2.7 ± 0.4	2.5 ± 0.5 *P* > 0.05	1.1 ± 0.3 *P* < 0.0001*	0.9 ± 0.3 *P* < 0.0001*

*P ≤ 0.05 was significant. ANOVA post hoc—the least significant difference option was used. Group data were compared against the CG group.

Notes: data are mean ± standard deviation. CG: control group; TG: thiamine 20 mg/kg + cisplatin group; TPG: TPP 20 mg/kg + cisplatin group; CG: control group; MDA: malondialdehyde; MPO: myeloperoxidase; tGSH: total glutathione; GST: glutathione S-transferase; GPx: glutathione peroxidase; GR: glutathione reductase; *n*: number of animals.
